# Effects of Dam and Sire Breeds on Lamb Carcass Quality and Composition in Pasture-Based Systems

**DOI:** 10.3390/ani13223560

**Published:** 2023-11-18

**Authors:** S. Maggie Justice, Elliot Jesch, Susan K. Duckett

**Affiliations:** 1 Department of Animal and Veterinary Sciences, Clemson University, Clemson, SC 29634, USA; mjustice@uada.edu; 2 Department of Food, Nutrition, and Packaging Sciences, Clemson University, Clemson, SC 29634, USA; ejesch@clemson.edu

**Keywords:** lamb, Texel, carcass composition, DXA, shear force, fatty acids

## Abstract

**Simple Summary:**

This research examined the use Texel or Southdown sires on Southdown or Suffolk dams to improve carcass quality and muscle composition in lambs produced on pasture-based systems. Texel-sired lambs had heavier carcasses, larger ribeye areas and individual muscle weights from the loin and leg. Dual energy X-ray absorptiometry was used to rapidly rank carcasses for leanness by dam and sire breeds. Texel-sired lambs had lower, more desirable, rank for carcass leanness and less total carcass fat. Individual muscles of the loin and leg from the various breed combinations were evaluated for fatty acid composition and tenderness. Dam and sire breed influenced fatty acid composition of the muscles. The semitendinosus muscle had the highest total fatty acid content and lowest ratio of n-6 to n-3 fatty acids. Overall, muscles from these lambs finished on pasture with limited grain supplementation were very lean, with high concentrations of polyunsaturated fatty acids and a ratio of n-6 to n-3 fatty acid of below 4:1, which is considered beneficial for human health and reduction of coronary heart disease. The use of Texel sires in pasture-based systems improved carcass leanness and muscle PUFA concentrations without altering tenderness.

**Abstract:**

This study explored the impacts of sire and dam breed on carcass quality and composition in a pasture-based system and the use of DXA to rapidly rank carcasses for leanness. Southdown (SD) and Suffolk (SF) ewes were mated to Texel (TX) or SD rams to produce seventy-nine lambs. Lambs were raised on pasture-based systems with limited grain supplementation. Lamb birth weight was greater (*p* < 0.01) for TX, regardless of dam breed. Lambing rate was lower (*p* < 0.01) for SD than SF ewes. Circulating myostatin concentrations were greater (*p* < 0.05) on d 42 than d 75 or d 110 but did not differ by sire breed. Texel-sired lambs had greater (*p* < 0.01) carcass weight, ribeye area and quality grade compared to SD-sired. Total and primal fat mass as predicted from DXA was higher (*p* < 0.05) in carcasses from SD than TX sires. Muscles from TX lambs had greater (*p* < 0.05) polyunsaturated fatty acid (PUFA) composition than SD-sired. Shear force values were influenced (*p* < 0.01) by dam breed, muscle cut and postmortem age but not by sire breed. The use of TX sires in pasture-based systems improved carcass leanness and muscle PUFA concentrations without altering tenderness.

## 1. Introduction

Lamb per capita consumption in the USA is low due to excess fat on lamb carcasses and consumer perceptions that lamb meat is high in saturated fat [1,2]. Consumers are looking for healthier food options including leaner meat products [3]. The industry is responding to consumer needs by producing leaner lambs through genetics [4]. This is often achieved through targeting genes that alter specific muscling traits with use of terminal sire breeds [5]. Texel sheep are known for their increased muscling, which is sometimes referred to as doubling muscling. This increased muscle mass is due to a G to A transition in the 3′ UTR of myostatin (MSTN) which then creates a target site for miRNAs to inhibit translation [6]. Studies have shown the advantages of using Texel sires to increase muscle and decrease fat on carcasses with feedlot finishing [7,8]; however, limited information is available on the impact of terminal sire breeds in pasture-finishing systems.

Production of lean lamb is important for the industry to remain competitive in the marketplace. Current grading systems were developed in 1960s when lambs were fatter and had higher yield grades. For the lamb industry to advance, the ability to accurately and quickly estimate carcass composition is imperative to meet these industry goals. Carcass yield grade and retail cut yield are based solely on fat thickness and no measures of muscle size and mass are included in the current USA grading system [2,9,10]. The use of terminal sire breeds produces leaner, heavier muscled lambs that predominantly fall into yield grade 1 and 2. Imaging technology has been shown to be accurate in carcass composition predictions [11,12]. One of these technologies is dual energy X-ray absorptiometry (DXA), which was developed to measure human body composition using X-rays at two different energies. DXA technology was first used in livestock around 1996 and has since shown potential to accurately predict whole carcass composition [13]. Therefore, the objectives of this study were to explore: (1) the impacts of sire and dam breed on growth, carcass composition and meat quality in a pasture-based system, and (2) the use of DXA to rapidly assess carcass or primal fat percentage, to rank these lambs on leanness.

## 2. Materials and Methods

### 2.1. Design

All animal experimental procedures were reviewed and approved by the Clemson University Institutional Animal Care and Use Committee (AUP 2018-049). Southdown (SD; n = 25) and Suffolk (SF; n = 25) multiparous ewes were mated to Texel (TX; Texel Muscled, GeneSeek) or Southdown (SD) rams. All lambs (n = 79) were raised by the dam on pasture and weaned at 75 d of age. Lambs were individually weighed at 0800 AM every 14 d during the pre-weaning phase. After weaning, wether lambs were finished on novel fescue pasture and supplemented with a high-energy feed. Wether lambs were individually weighed at 0800 AM prior to supplementation at 28-d intervals until they reached 57 kg. Wether lambs (n = 41) were fasted overnight and then transported to the Clemson University Meat Lab for humane slaughter. Weights of the carcasses were collected at the end of slaughter (hot carcass weight) and after chilling for 24 h at 2.2 °C (chilled carcass weight). Each carcass was split in half and then cut into four primal cuts, shoulder, rack, loin and leg [14], and then used for DXA scanning. The right half of the carcass was ribbed at the 12/13th rib and standard carcass variables were measured (USDA, 1992).

### 2.2. Myostatin

Circulating myostatin (MSTN) concentrations were measured at d 42, 75 and 110 after birth. Blood samples were collected via venipuncture and analyzed for MSTN using an enzyme-linked immunosorbent antibody (AbClonal, Woburn, MA, USA). The intra-assay variance was 6.78% and inter-assay variance was 10.87%.

### 2.3. DXA Analysis

Carcass body composition analysis was performed using Dual X-ray Absorptiometry (DXA) on a Hologic Discovery QDR Series (Hologic, Inc., Bedford, MA, USA) densitometer. Previous research had shown that the use of cold carcasses was superior to hot carcasses and that primals could be used to predict the whole carcass fat percentage [15].

### 2.4. Carcass Dissection

At the completion of the DXA scans, the left side was then used for individual muscle dissection and weighing. Each primal [14] was weighed and then the major muscles were dissected from the primal cuts and weighed. The major muscles taken from each primal were: longissimus muscle (LM; from rack and loin), gluteus medius (GM; from leg), biceps femoris (BF; from leg), semitendinosus (ST; from leg), semimembranosus (SM; from leg), adductor (AD; from leg), and quadriceps femoris (QF; from leg).

### 2.5. Fatty Acids

Muscle samples (LM, GM, SM and ST) were lyophilized and transmethylated according to the method of Park and Goins [16]. Fatty acid methyl esters (FAME) were analyzed by gas chromatography using a TR-FAME (Thermo Scientific, Thermo Fisher, Waltham, MA, USA) capillary column. Quantification of fatty acids in each sample was accomplished by adding an internal standard, methyl tricosanoic (C23:0), during methylation, and expressed as a weight percentage.

### 2.6. Warner–Bratzler Shear Force

The individual muscles of the LM, GM and SM were cut into 2.54 cm thick chops and randomly assigned to one of three postmortem aging treatments (d 1, 3 or 6). Chops were vacuum packaged and maintained at 4 °C until their assigned aging time, then frozen at −20 °C for subsequent shear force analyses according to AMSA [17]. Chops were thawed at 4 °C for 24 h and then broiled (BBQ Guys, Baton Rouge, LA, USA) to 71 °C, a medium degree of doneness, using thermocouples and a temperature logger. Chops were weighed before and after cooking to estimate cooking loss. After cooling, six cores (1.27 cm diameter) were removed from each chop for Warner–Bratzler shear force analyses (Standard shear force model 2000; G-R Manufacturing, Manhattan, KS, USA).

### 2.7. Statistical Analyses

Data were analyzed using SAS 9.4 (SAS Inst. Inc., Cary, NC, USA) using a mixed model with dam breed, sire breed or their interaction in the model. For circulating myostatin concentrations, a repeated measures analysis (GLIMMIX) was used that also included day and all interactions with dam and sire breed. For shear force and fatty acids, muscle was also included in the model with dam breed, sire breed and all interactions. Correlations among variables were calculated using the correlation procedure of SAS. Regression analysis was used to compare the relationship of DXA scans of the whole carcass half and DXA scans of each individual primal. Significance was determined at *p* < 0.05.

## 3. Results

A total of 79 lambs (158% lamb crop) was born in this study, comprising 38 female and 41 male lambs (Table 1). Lambing rate was greater (*p* < 0.05) for Suffolk dams than Southdown dams but did not differ (*p* > 0.05) by sire breed. Lamb birth weight was greater (*p* < 0.01) for Texel-sired compared to Southdown-sired lambs. Weaning weight tended to be greater (*p* < 0.10) for Texel-sired than Southdown-sired lambs. Growth rate was similar between all lambs except for d 14 to 28 when lambs born to Southdown dams had higher (*p* < 0.05) average daily gain compared to lambs from Suffolk dams.

**Figure 1:**
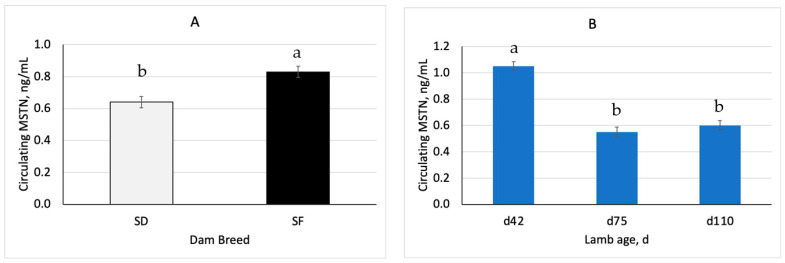
Effects of dam breed (**A**) and sire breed on circulating myostatin (MSTN) concentrations during growth (**B**). Dam breeds: SD = Southdown and SF = Suffolk. Sire breeds: TX = Texel and SD = Southdown. ^ab^ Means with uncommon superscripts differ (*p* < 0.05).

Circulating myostatin concentrations were measured by ELISA and values are shown in Figure 1. Myostatin concentration differed by dam breed (*p* = 0.0002) and animal age (*p* < 0.0001). Sire breed did not alter myostatin concentrations and all interactions were non-significant (*p* > 0.22). Myostatin concentrations were greater (*p* < 0.01) on d 45 than d75 or 110, regardless of sire or dam breed. Lambs born to Southdown ewes had lower (*p* < 0.01) myostatin concentrations than lambs born to Suffolk ewes.

Carcass characteristics and individual muscle weights of wethers by dam and sire breeds are shown in Table 2. Hot and chilled carcass weights were greater (*p* < 0.01) for Texel-sired than Southdown-sired lambs and for lambs born to Suffolk dams compared to Southdown dams. Dressing percentage and ribeye area were greatest (*p* < 0.05) for Suffolk × Texel lambs than other breed combinations. Flank streaking, conformation and quality grade scores were higher (*p* < 0.05) for Texel-sired lambs compared to Southdown-sired lambs. Suffolk × Texel carcasses had heavier (*p* < 0.05) rack, leg and total primal weight than other breed combinations; however, these changes were related to differences in carcass weight and when expressed on a percentage basis did not differ (*p* > 0.05). Lambs born to Southdown dams had a lower (*p* < 0.05) percentage of weight in leg primals compared to Suffolk dams. Suffolk × Texel lambs had greater (*p* < 0.05) longissimus, semitendinosus and semimembranosus muscle weights than other breed combinations.

Predicted total fat percentage of carcasses, all four primal cuts, rack and shoulder cuts from DXA scans differed (*p* < 0.05) by dam and sire breed but no interactions were observed (*p* > 0.05). Total carcass and primal fat content as measured by DXA was greater (*p* < 0.05) for Southdown-sired than Texel-sired lambs and for lambs from Southdown dams than Suffolk dams (Table 3). The percentage of fat in the loin, rack and leg was lower (*p* < 0.05) for Texel-sired lambs than Southdown-sired lambs. Lambs from Southdown dams had greater (*p* < 0.05) fat content in the rack and shoulder than Suffolk dams. Total primal fat percentage and total carcass fat percentage had a high agreement (r = 0.98), indicating that DXA measurements could be taken on vacuum packaged primals instead of carcass or side to facilitate scanning and food safety parameters. The leg and shoulder primals had the highest (r = 0.78 and 0.79, respectively) correlations with total carcass fat percentage; whereas the rack and loin had lower correlations (r = 0.60 and 0.59, respectively) with total carcass fat mass. Ranking carcasses for leanness based on DXA scans for total carcass fat percentage showed that sire breed altered (*p* < 0.05) leanness of the carcasses, with Texel-sired having leaner carcasses than Southdown-sired. Ranking on carcass yield grade did not show any differences (*p* > 0.05) in sire or dam breeds, which demonstrates its inability to separate carcasses for leanness in pasture-finished lambs. Stepwise equations were developed to predict carcass fat percentage using DXA shoulder fat percentage which explained over 60% of the variation (carcass fat, % = 9.84 + 0.63 × DXA shoulder fat percentage; r^2^ = 0.62).

All interactions between muscle and dam or sire breed were non-significant for proximate composition (Table 4). Total lipid content was greater (*p* < 0.05) for Texel-sired lambs than Southdown-sired. Moisture content was highest (*p* < 0.05) for SDTX and lowest for SDSD. Moisture content of the individual muscles was greater (*p* < 0.05) for SM than GM, LM, or ST. Total lipid content was greater (*p* < 0.05) for ST than GM, LM or SM.

Fatty acid composition by sire and dam breed across muscle from the loin and leg are shown in Table 5. All two-way and three-way interactions between muscle and dam or sire breed were non-significant. Stearic acid concentration was highest (*p* < 0.05) in muscles from SFSD and lowest (*p* < 0.05) for SFTX. For the SDTX lamb muscles, trans-11 vaccenic (C18:1 t11) acid concentration was lowest (*p* < 0.05) and ratio of n-6 to n-3 PUFA was highest (*p* < 0.05) compared to other dam × sire breed combinations. For SFTX lamb muscles, linolenic acid, EPA, and total n-3 PUFA concentrations were greater (*p* < 0.05) than other dam × sire breed combinations. Arachidic (C20:0) acid concentration was greatest (*p* < 0.05) and CLA lowest (*p* < 0.05) for SDTX and SFSD.

Southdown dams produced lambs with greater (*p* < 0.05) myristic (C14:0), palmitic (C16:0) and saturated fatty acid concentrations in the four muscles examined than Suffolk. Palmitoleic (C16:1) acid was greater (*p* < 0.05) and cis-11 vaccenic (C18:1) acid concentration was lower (*p* < 0.05) in muscles from lambs born to Southdown dams. Linoleic (C18:2) acid and total n-6 polyunsaturated fatty acid (PUFA n-6) concentrations were lower (*p* < 0.05) in muscles from lambs born to Southdown dams. Docosahexaenoic (C22:6; DHA) acid concentration was greater (*p* < 0.05) in muscles from lambs born to Southdown dams.

Texel-sired lambs had greater (*p* < 0.05) concentrations of linoleic acid, arachidonic (C20:4 n-6) acid and total n-6 PUFA in muscles compared to Southdown-sired lambs. Oleic (C18:1 c9) acid, cis-11 vaccenic acid, margaric (C17:0) acid, SFA, MUFA and OCFA concentrations were lower (*p* < 0.05) in Texel-sired than Southdown-sired lambs. Total n-3 PUFA, eicosatrienoic (C20:3 n-3) acid, EPA, docosapentaenoic (C22:5 n-3, DPA) and DHA concentrations were greater (*p* < 0.05) for Texel-sired than Southdown-sired lambs. Total fatty acid content of the muscles was lower (*p* < 0.05) for Texel-sired than Southdown-sired lambs.

Fatty acid composition of individual muscles examined in this study showed that many differences existed (Table 6). Total fatty acid content was higher (*p* < 0.05) for LM and ST than GM and SM. Saturated fatty acid concentration was lowest (*p* < 0.05) in SM and MUFA concentration was lowest (*p* < 0.05) in GM. Concentrations of n-6 PUFA were greater (*p* < 0.05) for GM and SM and n-3 PUFA concentrations were greater (*p* < 0.05) for GM than LM and ST. The ratio of n-6 to n-3 PUFA was lowest (*p* < 0.05) for ST muscle.

**Figure 2:**
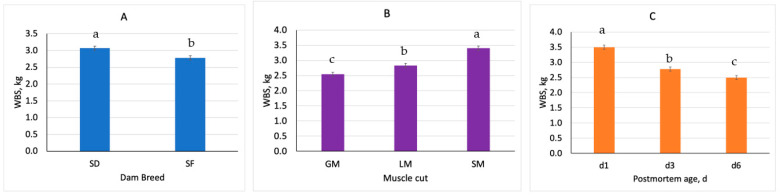
Effects of dam breed (**A**), muscle location (**B**) and post-mortem aging time (**C**) on Warner–Bratzler shear force (WBS) values in 3 muscles. Dam breeds: SD = Southdown and SF = Suffolk. Muscle location: GM = gluteus medius (leg), LM = longissimus (rack and loin), SM = semimembranosus (leg). ^abc^ Means with uncommon superscripts differ (*p* < 0.05).

All interactions between dam breed, sire breed, muscle and postmortem aging time were non-significant. Warner–Bratzler shear force values were lower (*p* < 0.05) in muscles of lambs born to Suffolk than Southdown breeds (Figure 2A). Sire breed did not alter Warner–Bratzler shear force values. The GM had the lowest (*p* < 0.05) shear force value, and the SM had the highest (*p* < 0.05) shear force value (Figure 2B). Postmortem aging reduced (*p* < 0.05) Warner–Bratzler shear force values at each time point (*p* < 0.05), with the greatest change between d 1 and d 3 (Figure 2C).

## 4. Discussion

Terminal sire breeds are often utilized to improve carcass leanness and muscle mass, but they may alter lambing and production characteristics [18,19]. Our results show that sire breed did not impact lambing rate but did increase birth weight by 15% for Texel-sired lambs. Lamb growth rates were similar during preweaning growth, except between d 14 and d 28 when lambs from Southdown dams grew faster than Suffolk dams. Weaning weight tended to be higher for Texel-sired lambs. Others [19] have shown that lamb number per ewe and litter weights were lower for Texel sires compared to more prolific breeds like the Romanov. Freking and Leymaster (2004) reported that Texel-sired lambs had slower growth from d70 to 140 compared to Dorset, Romanov or Montadale-sired lambs. Suffolk offspring have been shown to have leaner growth than other breeds. including Southdown lambs [20].

The enhanced muscle phenotype of the Texel is related to a single nucleotide polymorphism (c. *1232 G > A) in the myostatin gene that provides a binding site for miR-1 and miR-206 [6]. Tellam et al. [21] reported that lambs sired by an F2 Texel ram had approximately one-third lower circulating MSTN concentrations compared to wild-type sheep. Unfortunately, the timing of the blood sample collection in that study was not described in the paper. We observed that circulating myostatin concentrations differed over time, with younger suckling lambs (d 42) having higher values than weaned (d 75 and 110) lambs; however, there was no difference between sire breeds.

Texel-sired lambs had greater carcass weights, quality and individual muscle weights than Southdown-sired lambs. Dressing percentage, ribeye area, rack weight and leg weight were greater for Suffolk × Texel lambs than other breed combinations. Others have reported that Texel lambs have a similar carcass composition to Suffolk lambs but are considered more compact [22]. The Texel breed is known for its superior muscling phenotype due to a myostatin mutation [4]. Because of this mutation, Texel and Texel cross lambs have been shown to have improved carcass lean, with less fat in various locations throughout the carcass [8]. Overall, the use of the Texel as a terminal sire breed in pasture-finishing systems with limited supplementation did improve carcass weight, muscling and leanness, with similar growth performance measures.

All lamb carcasses in this study had yield grades of 1 and 2 and were graded as Prime and Choice, which is similar to national averages [23]. Therefore, we examined the use of the DXA technology to rapidly scan the carcass or primal cuts to rank carcasses on leanness. Our results show that percentage of fat could be predicted in the carcass or the four major primals with a high correlation (r = 0.98). Texel-sired lambs had a lower, more desirable, rank for DXA carcass leanness and ribeye area. Others [24] have reported similar errors in current USDA grading methods for segregating carcass leanness. The use of technology in analyzing carcass composition can help to improve these carcass composition analyses methods [25,26,27]. By utilizing DXA technology, studies have found that other factors such as muscle mass are more useful in predicting carcass composition in lean lambs than fat depth alone [26,28]. Connaughton et al. [29,30] have reported that DXA technology can be used in the abattoir at chain speed to predict carcass fat percentages with high repeatability.

Limited research is available about changes in fatty acid composition of muscles from the dam and sire breeds used in this study on pasture-based systems. Total fatty acid content of the muscles was lower for Texel-sired than Southdown-sired lambs. Texel-sired lambs had greater concentrations of total n-6 PUFA and n-3 PUFA, and lower concentrations of SFA, MUFA and OCFA. Previous research comparing sire breeds also showed that Texel-sired lambs had higher PUFA and both n-6 and n-3 concentrations than lambs from other sires [31]. Snowder and Duckett [32] also reported differences in fatty acid composition of LM by different sire breeds, Dorper and Suffolk, when feedlot finished. Finishing regimes alter fat deposition and fatty acid composition of muscle tissues in sheep [1,33], goats [33] and cattle [34,35].

The semitendinosus muscle had the highest total lipids, total fatty acid content, and lowest ratio of n-6 to n-3 PUFA in this study. In contrast, the semitendinosus muscle is considered one of the leanest muscles in beef carcasses finished on pasture [35]. Saturated fatty acid concentration was lowest in SM and MUFA concentration was lowest in the GM. Relationships between SFA concentration and total fatty acid content are high in beef [35] and our results in sheep are similar, with leaner muscles having a lower concentration of SFA. Omega-6 PUFA concentrations were greater for GM and SM, whereas, n-3 PUFA concentrations were greater for GM than LM and ST. Overall, the results demonstrate that carcasses of the different sire and dam breeds used in this study were lean (<1.8 g/100 g muscle), with high concentrations of PUFA (6.4 to 8.9% PUFA n-6 and 2.0 to 2.5% PUFA n-3). The ratio of n-6 to n-3 fatty acids was below 4:1, the upper level recommended for human health and reduction in coronary heart disease [36,37], for all muscles and breed combinations for lambs finished on pasture with limited grain supplementation.

Tenderness of the lamb muscles as measured by Warner–Bratzler shear force showed that the SM muscle was toughest and GM the most tender. Sire breed did not influence tenderness but dam breed did. Others [8,38] have reported that the use of Texel sires did not alter tenderness. In contrast, previous research evaluating different sire breeds found that Texel-sired and Suffolk-sired lamb had higher shear force values in the LM and GM but not the SM or ST, compared to Southdown-sired lambs [31]. Postmortem aging of all muscles improved tenderness, with the greatest change in shear force value from d 1 to d 3 (72% of total improvement); however, aging to 6 d postmortem further improved tenderness but the magnitude of the change was lower (28% of total improvement) from d 3 to d 6.

## 5. Conclusions

The use of a terminal sire breed like the Texel for different ewe populations improved muscle growth and carcass quality without altering growth performance or tenderness. Dual energy X-ray absorptiometry can be used to rapidly rank leanness of lamb carcasses or primals. Lamb muscles were lean, with high concentrations of PUFA, and a desirable n-6 to n-3 ratio.

## Figures and Tables

**Table 1:** Effects of dam and sire breed on ewe lambing rate, birth weight and lamb growth performance.

Dam Breed ^1^	SD	SD	SF	SF		*p*-Levels		
Sire Breed ^2^	SD	TX	SD	TX	SE	Dam	Sire	Int
n	15	16	24	24				
Lambing rate	1.67	1.56	1.92	1.82	0.41	0.0085	0.27	0.95
Lamb sex	1.67	1.50	1.58	1.5	0.50	0.72	0.28	0.72
Live lambs	1.00	0.94	0.96	1.00	0.16	0.85	0.85	0.19
Birth weight, kg	4.36	5.04	4.24	4.86	0.90	0.47	0.0022	0.88
Wean weight, kg	29.10	31.18	28.49	29.78	4.13	0.30	0.086	0.69
ADG, kg/d								
0–14	0.34	0.38	0.37	0.35	0.099	0.90	0.61	0.27
14–28	0.31	0.35	0.26	0.24	0.16	0.027	0.68	0.51
28–42	0.34	0.35	0.35	0.36	0.18	0.72	0.75	0.97
42–56	0.39	0.36	0.34	0.35	0.20	0.51	0.82	0.69
56–75	0.38	0.40	0.41	0.46	0.20	0.37	0.46	0.74
overall	0.33	0.35	0.32	0.33	0.20	0.33	0.29	0.81

^1^ Dam breeds: SD = Southdown and SF = Suffolk. ^2^ Sire breeds: TX = Texel and SD = Southdown.

**Table 2:** Effects of dam and sire breed on wether lamb carcass quality, composition and muscle mass.

Dam Breed ^1^	SD	SD	SF	SF		*p*-Levels		
Sire Breed ^2^	SD	TX	SD	TX	SE	Dam	Sire	Int
n (wethers)	9	9	12	11				
Birth wt, kg	4.32	5.32	4.29	5.14	0.90	070	0.0022	0.80
Wean wt, kg	29.70	31.72	28.64	30.29	4.54	0.39	0.20	0.90
Final wt, kg	50.60	51.72	52.90	55.45	5.35	0.084	0.29	0.68
Pre-wean adg, kg/d	0.34	0.35	0.32	0.34	0.053	0.36	0.46	0.93
Post-wean adg, kg	0.11	0.11	0.15	0.14	3.17	0.0001	0.83	0.50
Age at harvest, d	255.4	255.1	247.2	253.1	19.29	0.41	0.65	0.62
Carcass traits								
Hot carcass wt, kg	27.95	28.30	29.17	31.34	1.91	0.0012	0.045	0.14
Dressing percent	53.27 ^b^	52.61 ^b^	52.93 ^b^	55.08 ^a^	0.017	0.055	0.18	0.013
Chill carcass wt, kg	27.1	27.4	28.4	30.7	1.84	0.0004	0.028	0.10
Fat thickness, cm	0.14	0.17	0.16	0.16	0.041	0.70	0.31	0.30
Yield grade	1.83	2.10	2.02	2.02	0.41	0.70	0.31	0.30
Ribeye area cm ^2^	2.32 ^c^	2.70 ^b^	2.53 ^bc^	3.33 ^a^	0.27	0.0001	0.0001	0.022
Flank streaking ^3^	18.0	19.1	18.5	19.3	1.40	0.49	0.037	0.74
Conformation ^3^	17.8	19.2	18.2	20.1	0.90	0.024	0.0001	0.50
Quality grade ^3^	17.9	19.2	18.3	19.7	0.88	0.086	0.0001	0.94
Primals								
Shoulder, kg/side	3.22	3.14	3.20	3.55	0.33	0.072	0.22	0.54
Rack, kg/side	1.46 ^b^	1.46 ^b^	1.45 ^b^	1.68 ^a^	0.14	0.025	0.014	0.015
Loin, kg/side	1.51	1.49	1.51	1.71	0.22	0.12	0.19	0.13
Leg, kg/side	4.50 ^c^	4.71 ^bc^	4.80 ^b^	5.48 ^a^	0.29	0.0001	0.0001	0.017
Total, kg/side	10.69 ^b^	10.80 ^b^	10.97 ^b^	12.42 ^a^	0.81	0.0008	0.0044	0.014
Shoulder, %	30.08	29.03	29.17	28.55	1.62	0.19	0.11	0.68
Rack, %	13.60	13.52	13.21	13.54	0.73	0.42	0.60	0.38
Loin,%	14.03	13.78	13.74	13.79	1.36	0.75	0.81	0.74
Leg, %	42.29	43.67	43.87	44.12	1.56	0.047	0.11	0.26
Muscle wt, g/side								
Longissimus	646.55 ^b^	670.92 ^b^	677.78 ^b^	846.43 ^a^	98.01	0.0020	0.0037	0.026
Semitendinosus	151.58	160.28	172.07	197.36	13.41	0.0001	0.0003	0.059
Gluteus medius	305.03	344.45	321.08	383.99	27.79	0.0033	0.0001	0.19
Biceps femoris	406.40	470.41	483.24	543.39	42.89	0.0001	0.0001	0.89
Adductor	166.87	200.37	194.26	234.43	18.15	0.0001	0.0001	0.56
Quadriceps femoris	555.48	595.86	601.76	675.76	64.28	0.0038	0.0081	0.42
Semimembranosus	415.51 ^b^	421.30 ^b^	444.10 ^b^	507.68 ^a^	35.18	0.0001	0.0037	0.014
Total excised	2647.4 ^c^	2863.6 ^b^	2894.3 ^b^	3389.0 ^a^	215.49	0.0001	0.0001	0.049

^1^ Dam breeds: SD = Southdown and SF = Suffolk. ^2^ Sire breeds: TX = Texel and SD = Southdown. ^3^ Flank streaking, conformation and quality grade code: 16 = Good^+^, 17 = Choice−, 18 = Choice°, 19 = Choice^+^, 20 = Prime−, 21 = Prime°, 22 = Prime+. ^abc^ Means without common superscripts in the same row differ (*p* < 0.05).

**Table 3:** Effects of dam and sire breed on wether lamb carcass and primal composition as measured using dual X-ray absorptiometry.

Dam Breed ^1^	SD	SD	SF	SF		*p*-Levels		
Sire Breed ^2^	SD	TX	SD	TX	SE	Dam	Sire	Int
n (wethers)	9	9	12	11				
Total carcass fat %	31.15	28.63	29.03	27.54	2.38	0.04	0.012	0.50
Total primal fat %	31.53	28.54	29.30	27.14	2.64	0.037	0.0041	0.63
Leg fat %	29.82	27.02	26.45	27.66	3.79	0.26	0.51	0.10
Loin fat %	35.80	30.28	33.76	29.30	4.60	0.31	0.0016	0.72
Rack fat %	30.36	26.78	29.18	22.94	3.27	0.021	0.0001	0.21
Shoulder fat %	32.46	30.81	31.45	27.13	2.62	0.0077	0.0009	0.12
Carcass lean rank ^3^	30.50	19.11	21.77	14.73	11.08	0.070	0.013	0.054
Yield grade rank ^3^	16.88	24.78	22.0	19.73	12.13	0.99	0.47	0.20
Ribeye area rank ^3^	32.75	21.00	26.08	6.45	7.30	0.0001	0.0001	0.098

^1^ Dam breeds: SD = Southdown and SF = Suffolk. ^2^ Sire breeds: TX = Texel and SD = Southdown. ^3^ Carcass lean, yield grade and ribeye area rank: carcasses were ranked (1 being most desirable for the trait) from leanest, lowest yield grade and largest ribeye area. The values presented here are the average of each rank by dam and sire breed.

**Table 4:** Effects of dam and sire breed, and muscle location on muscle moisture and lipid content.

Dam Breed ^1^	SD	SD	SF	SF				*p*-Levels
Sire Breed ^2^	SD	TX	SD	TX	SEM	Dam	Sire	Int
n	41	41	41	41				
Moisture, %	74.07 ^c^	74.96 ^a^	74.45 ^b^	74.66 ^ab^	0.12	0.74	0.0001	0.0041
Total lipid, %	3.15	2.37	2.83	2.26	0.12	0.084	0.0001	0.40
Muscle ^3,4^	GM	LM	SM	ST	SEM	*p*-Level		
n	41	41	41	41				
Moisture, %	74.81 ^a^	74.55 ^a^	74.21 ^b^	74.57 ^a^	0.12	0.0043		
Total lipid, %	2.52 ^b^	2.50 ^b^	2.33 ^b^	3.26 ^a^	0.12	0.0001		

^1^ Dam breeds: SD = Southdown and SF = Suffolk. ^2^ Sire breeds: TX = Texel and SD = Southdown. ^3^ Muscle abbreviations (primal): GM = gluteus medius (leg), LM = longissimus (rack and loin), SM = semimembranosus (leg), and ST = semitendinosus (leg). ^4^ All two-way and three-way interactions between muscle and dam breed or sire breed were non-significant (*p* > 0.05). ^abc^ Means without common superscripts in the same row differ (*p* < 0.05).

**Table 5:** Effects of dam and sire breed on fatty acid composition of lamb muscles.

Dam Breed ^1^	SD	SD	SF	SF		*p*-Levels		
Sire Breed ^2^	SD	TX	SD	TX	SE	Dam	Sire	Int
n	41	41	41	41				
C14:0, %	2.26	2.22	1.91	2.16	0.57	0.025	0.23	0.11
C15:0, %	0.34	0.29	0.30	0.30	0.11	0.31	0.13	0.12
C16:0, %	22.05	21.91	21.34	21.23	1.52	0.0050	0.62	0.96
C16:1, %	1.53	1.53	1.35	1.38	0.21	0.0001	0.62	0.54
C17:0, %	0.55	0.50	0.51	0.47	0.11	0.090	0.0082	0.68
C18:0, %	18.35 ^b^	17.82 ^bc^	19.00 ^a^	17.52 ^c^	1.36	0.41	0.0001	0.028
C18:1t9, %	0.14	0.16	0.22	0.16	0.19	0.20	0.51	0.24
C18:1t10, %	0.16	0.10	0.10	0.16	0.21	0.88	0.97	0.077
C18:1t11, %	2.30 ^a^	1.95 ^b^	2.17 ^a^	2.15 ^a^	0.44	0.66	0.0090	0.024
C18:1c9, %	38.83	37.29	38.91	36.62	2.39	0.44	0.0001	0.32
C18:1c11, %	0.78	0.88	0.82	0.96	0.10	0.0001	0.0001	0.28
C18:2 c9,12, %	4.64	5.89	5.28	6.59	1.24	0.0009	0.0001	0.88
C18:3 c9,12,15, %	1.06 ^b^	1.01 ^b^	1.05 ^b^	1.16 ^a^	0.22	0.043	0.34	0.026
C20, %	0.10 ^b^	0.11 ^a^	0.11 ^a^	0.09 ^b^	0.020	0.40	0.44	0.0001
CLA, c9t11, %	0.56 ^a^	0.48 ^b^	0.49 ^b^	0.51 ^ab^	0.11	0.31	0.22	0.014
C20:2, %	0.04	0.05	0.06	0.07	0.059	0.080	0.14	0.69
C20:3, %	0.18	0.24	0.18	0.23	0.084	0.74	0.0001	0.50
C20:4, %	1.54	2.08	1.69	2.04	0.50	0.51	0.0001	0.26
C20:5, %	0.38 ^b^	0.38 ^b^	0.37 ^b^	0.48 ^a^	0.13	0.033	0.0061	0.013
C22:5, %	0.51	0.56	0.51	0.63	0.15	0.11	0.0005	0.14
C22:6, %	0.17	0.21	0.16	0.18	0.052	0.023	0.0003	0.56
Identified, %	96.47	95.69	96.55	95.13	1.94	0.44	0.0005	0.31
SFA, %	42.76	42.07	42.37	41.02	1.98	0.023	0.0014	0.29
OCFA, %	0.89	0.79	0.81	0.77	0.18	0.11	0.016	0.26
MUFA, %	40.36	38.82	40.25	38.00	2.41	0.23	0.0001	0.36
PUFA, n-6, %	6.41	8.27	7.2	8.94	1.75	0.0091	0.0001	0.81
PUFA, n-3, %	2.12 ^b^	2.16 ^b^	2.09 ^b^	2.46 ^a^	0.51	0.13	0.012	0.049
Ratio n-6:n-3	3.05 ^c^	3.84 ^a^	3.48 ^b^	3.74 ^ab^	0.73	0.15	0.0001	0.021
Total fatty acids, g/100g muscle	1.77	1.36	1.60	1.29	0.53	0.16	0.0001	0.58

^1^ Dam breeds: SD = Southdown and SF = Suffolk. ^2^ Sire breeds: TX = Texel and SD = Southdown. ^abc^ Means without common superscripts in the same row differ (*p* < 0.05).

**Table 6:** Fatty acid composition of individual muscles from lambs of the various sire and dam breeds utilized in this study.

Muscle ^1,2^	GM	LM	SM	ST	SEM	*p*-Level
n	41	41	41	41		
C14:0, %	2.14 ^b^	1.95 ^b^	2.06 ^b^	2.41 ^a^	0.091	0.0044
C15:0, %	0.32 ^b^	0.25 ^c^	0.30 ^b^	0.36 ^a^	0.016	0.0001
C16:0, %	20.84 ^c^	21.96 ^b^	20.95 ^c^	22.78 ^a^	0.24	0.0001
C16:1, %	1.40 ^b^	1.38 ^b^	1.50 ^a^	1.52 ^a^	0.033	0.0029
C17:0, %	0.51	0.50	0.50	0.54	0.018	0.18
C18:0, %	18.95 ^a^	18.73 ^a^	17.28 ^b^	17.74 ^b^	0.22	0.0001
C18:1t9, %	0.21 ^a^	0.11 ^b^	0.23 ^a^	0.12 ^b^	0.031	0.0052
C18:1t10, %	0.16 ^ab^	0.080 ^b^	0.091 ^b^	0.20 ^a^	0.033	0.028
C18:1t11, %	2.28	2.03	2.10	2.16	0.071	0.088
C18:1c9, %	35.93 ^b^	39.00 ^a^	38.07 ^a^	38.65 ^a^	0.38	0.0001
C18:1c11, %	0.86 ^b^	0.84 ^b^	0.91 ^a^	0.84 ^b^	0.016	0.0020
C18:2 c9,12, %	6.47 ^a^	5.10 ^b^	6.02 ^a^	4.81 ^b^	0.20	0.0001
C18:3 c9,12,15, %	1.19 ^a^	1.01 ^b^	1.07 ^b^	1.01 ^b^	0.035	0.0009
C20, %	0.11 ^a^	0.10 ^b^	0.11 ^a^	0.10 ^b^	0.0030	0.0001
CLA, c9t11, %	0.53	0.48	0.51	0.54	0.018	0.091
C20:2, %	0.049 ^b^	0.043 ^b^	0.099 ^a^	0.040 ^b^	0.0093	0.0001
C20:3, %	0.26 ^a^	0.20 ^b^	0.19 ^b^	0.18 ^b^	0.013	0.0001
C20:4, %	2.13 ^a^	1.69 ^b^	2.02 ^a^	1.52 ^b^	0.080	0.0001
C20:5, %	0.45 ^a^	0.38 ^bc^	0.44 ^ab^	0.34 ^c^	0.020	0.0004
C22:5, %	0.61 ^a^	0.52 ^b^	0.57 ^ab^	0.52 ^b^	0.024	0.027
C22:6, %	0.20 ^a^	0.16 ^b^	0.20 ^a^	0.16 ^b^	0.0083	0.0001
Identified, %	95.60 ^b^	96.49 ^a^	95.21 ^b^	96.54 ^a^	0.31	0.0043
SFA, %	42.04 ^b^	42.73 ^ab^	40.40 ^c^	43.03 ^a^	0.31	0.0001
OCFA, %	0.82 ^b^	0.74 ^c^	0.79 ^bc^	0.90 ^a^	0.030	0.0023
MUFA, %	37.33 ^b^	40.37 ^a^	39.56 ^a^	40.17 ^a^	0.38	0.0001
PUFA, n-6, %	8.91 ^a^	7.03 ^b^	8.33 ^a^	6.55 ^b^	0.28	0.0001
PUFA, n-3, %	2.45 ^a^	2.07 ^b^	2.28 ^ab^	2.03 ^b^	0.081	0.0008
Ratio n-6:n-3	3.64 ^ab^	3.41 ^bc^	3.84 ^a^	3.21 ^c^	0.12	0.0001
Total fatty acids, g/100 g muscle	1.38 ^b^	1.75 ^a^	1.09 ^c^	1.81 ^a^	0.084	0.0001

^1^ Muscle abbreviations (primal): GM = gluteus medius (leg), LM = longissimus (rack and loin), SM = semimembranosus (leg), and ST = semitendinosus (leg). ^2^ All two-way and three-way interactions between muscle and dam breed or sire breed were non-significant (*p* > 0.05). ^abc^ Means with uncommon superscripts in the same row differ (*p* < 0.05).

## Data Availability

Datasets generated from the current experiments are available from the corresponding author upon reasonable request.
